# Improving clinical care of patients in Nipah outbreaks: moving beyond ‘compassionate use’

**DOI:** 10.1016/j.lansea.2024.100527

**Published:** 2025-01-03

**Authors:** Md Zakiul Hassan, Amanda Rojek, Piero Olliaro, Peter Horby

**Affiliations:** a International Centre for Diarrheal Disease Research, Bangladesh (icddr, b), Dhaka, Bangladesh; b Pandemic Sciences Institute, Nuffield Department of Medicine, University of Oxford, Oxford, UK; c International Severe Acute Respiratory and Emerging Infection Consortium (ISARIC), University of Oxford, Oxford, UK; d University of Melbourne, Melbourne, Australia

**Keywords:** Nipah virus, Outbreak, Compassionate drug use, Epidemic, Pandemic, Southeast Asia, Clinical trials, Encephalitis, Therapeutics

## Abstract

The 2024 Nipah outbreak in Kerala, India—its fifth in six years—and the recurring annual outbreaks in Bangladesh underscore the persistent threat posed by the Nipah virus (NiV) in the region. With a high mortality rate, human-to-human transmission potential, and the widespread presence of *Pteropus* bats, the natural reservoir, NiV remains a significant epidemic threat. Despite being a WHO priority pathogen, there has been no systematic effort to improve patient care for NiVD, leading to consistently poor outcomes. Current care relies on supportive measures and the ‘compassionate use’ of unapproved drugs like ribavirin and remdesivir. Drugs used ‘off-label’ during outbreaks can become the ‘standard of care’ without robust evidence of their safety or efficacy, complicating the testing of new therapies and perpetuating uncertainty about their true effectiveness. To improve NiVD care, we propose four key strategies: 1) Enhance early case detection, 2) optimize supportive care to improve outcomes and create a standard for future trials, 3) adopt a syndromic approach centered on encephalitis, and 4) explore innovative trial designs tailored to low case numbers as an alternative to ‘compassionate use’. By integrating these strategies, healthcare systems in NiV-endemic regions will be better equipped to manage both current and future outbreaks.

## Introduction

In July 2024, Kerala, India, reported its fifth Nipah virus (NiV) outbreak in six years, following the death of a 14-year-old boy.^1^^,^^2^ NiV, a bat-borne paramyxovirus first identified in Malaysia in 1998, has caused approximately 734 human cases and 429 (58%) deaths across five countries.^3^ Its capacity for human-to-human transmission and the widespread presence of its natural host, the *Pteropus* bat, underscore the constant threat of larger epidemics.^4^

These recurrent NiV outbreaks in South and Southeast Asia, with high mortality and poor patient outcomes, highlight critical gaps in Nipah virus disease (NiVD) clinical management. Despite being a World Health Organization (WHO) priority pathogen for research and development, there has been no systematic effort to improve NiVD patient care, leading to consistently poor outcomes.^5^ No approved vaccines or treatments exist. Care relies on supportive measures and occasional use of repurposed, unapproved drugs, often hindered by delayed diagnosis due to nonspecific symptoms and the lack of rapid tests. This reactive approach, lacking targeted interventions and evaluation, results in poor outcomes and highlights the need for a systematic, pragmatic approach—not only to improve outcomes in current outbreaks but also to better prepare for future, potentially more widespread epidemics.

## Compassionate use of drugs against NiVD

“Compassionate use” is the administration of medications that are not licensed for the specific indication outside of a clinical trial under the premise that they may be efficacious in the absence of valid alternatives. Ribavirin, a nucleoside analog prodrug approved for hepatitis C and respiratory syncytial virus, was used in NiVD outbreaks in Malaysia, Singapore, and India (Table 1).

**Table 1:** Clinical reports of compassionate drug use during Nipah virus outbreaks.

Reference	Outbreak	Patient who received treatment	Dose and route (n)	Outcome	Reported adverse effects
**Ribavirin**					
Chong 2001	Malaysia 1998–99	Total: 194, ribavirin group (lab-confirmed NiVD): 140, Control group (patient managed prior to availability of ribavirin/refused to take ribavirin): 54	IV (n = 128): Loading dose: 30 mg/kg initially, followed by 16 mg/kg every 6 h for 4 days, then 8 mg/kg every 8 h for 3 days PO (n = 12): Day 1: 2 g, Days 2–4: 1.2 g TDS, Days 5 and 6: 1.2 g BD, for an additional 1–4 days: 0.6 g BD	Mortality: ribavirin group: 32% (45/140), control group: 54% (29/54), p = 0.01; ventilatory support: Ribavirin: 57% (80/140), control: 54% (29/54), p = 0.14; Survivors without neurological deficits: ribavirin: 52% (73/140), control: 41% (22/54), p = 0.17	No statistically significant difference in incidence of anemia and bilirubinemia between treatment and control group
Goh 2000	Malaysia 1998–99	73 of 94 laboratory-confirmed NiVD cases	Either PO or IV in severely ill patients, dose not available	No significant difference in outcome with ribavirin treatment	Not available
Chong 2003	Malaysia 1998–99	Two relapsed and one late-onset encephalitis NiVD cases	PO for one week for one case, dose not available for the other two cases	All recovered. One case had persistent mild weakness of the right upper limb	Not available
Thulaseedaran 2018	Kerala, India, 2018	6 laboratory-confirmed NiVD cases	1000 mg (in 6 divided doses) for 14 days, route not available	2/6 survived, recovered without any sequalae	Not available
Banerjee 2019	Kerala, India, 2018	Post exposure prophylaxis (within 72 h of exposure) to eight HCWs, none developed NiVD	1000 mg TDS for 14 days; route not available	None of the eight HCWs developed NiVD and all survived	None completed course. 6/8 had transient increase in bilirubin and/or fall in haemoglobin; 6/8 experienced symptoms of fatigue, headache, nausea, dry mouth and palpitations
Gayathri 2019	Kerala, India, 2018	6 of 10 laboratory-confirmed NiVD cases received ribavirin	Not available	2/6 survived	Not available
Kumar 2019	Kerala, India, 2018	Five laboratory-confirmed NiVD cases	Not available	All died	Not available
Chandni 2020	Kerala, India, 2018	12 laboratory-confirmed NiVD cases (six treated, six untreated)	2 g IV loading followed by 1 g IV QDS for 4 days, then 500 mg PO QDS for 6 days	Ribavirin group: 4/6 died, untreated: 6/6 died, p = 0.4545)	Not available
Warrior 2020	Kerala, India, 2019	One laboratory-confirmed NiVD case	Not available, also treated with immunoglobulins	Survived and recovered fully from encephalitis after 51 days	Not available
Pallivalappil 2020	Kerala, India, 2018	8 of 19 laboratory- confirmed NiVD cases	1200 mg/day (in 6 divided doses) for 14 days, route not available	2 survived	Not available
Kumar 2024	Kerala, India, 2023	1 of 6 laboratory-confirmed NiVD cases received ribavirin	15 mg/kg, QDS, for 10 days, route not available	Recovered	Not available
**Remdesivir**					
Kumar 2024	Kerala, India, 2023	3 of 6 laboratory-confirmed NiVD cases	IV**:** 200 mg loading dose followed by 100 mg maintenance dose for 12 days	All three recovered. Case-1: became afebrile and asymptomatic after 2 days of initiation of remdesivir. All samples tested negative for NiV by POD 16 (12 days of remdesivir). Case-2: became asymptomatic after 1 day of remdesivir. All samples tested negative for NiV by POD 18 (12 days of Remdesivir). Case-3: became asymptomatic 7 days after the initiation of remdesivir treatment. All samples tested negative by POD 14 (12 days of remdesivir)	Not available

Note: NiVD, Nipah virus disease; IV, intravenous; PO, oral administration; BD, twice daily; TDS, three times daily, and QDS, four times daily; POD, post-onset day.

The open-label study conducted in Malaysia during the 1998–99 outbreak, which reported improved outcomes with ribavirin, was observational in nature and utilized historical controls.^6^ Observational studies are subject to significant potential biases and confounding that limit the strength of the conclusions.^7^ Thus, reports of improved outcomes in an observational study represents weak evidence that should not be used to develop treatment guidelines. Whilst studies in animal models showed that ribavirin delayed the time to death, it did not prevent death or symptoms after NiV-M inoculation in hamsters compared to untreated controls.^8^^,^^9^

Remdesivir (GS-5734), a nucleotide analog prodrug approved for COVID-19, was used in the 2023 Kerala outbreak.^10^ During the 2018 Kerala outbreak, the experimental m102.4 monoclonal antibody (mAb) was imported for treating NiVD patients on compassionate grounds—though it was not used as the outbreak had ended by then, nor was it used in the 2019 Kochi outbreak, as there were no new confirmed cases, and the patient was in the recovery phase when NiV infection was confirmed.

The Department of Health and Family Welfare, Government of Kerala, India, have issued guidelines for managing Nipah virus disease, recommending the use of ribavirin, remdesivir, favipiravir (a viral RNA-dependent RNA polymerase inhibitor), and the monoclonal antibody m102.4 on a compassionate basis during outbreaks.^11^ In contrast, the National Guideline for the Management, Prevention, and Control of Nipah Virus Infection, including Encephalitis, in Bangladesh, focuses primarily on supportive care and does not endorse any Nipah-specific treatments.^12^ Despite accounting for over half of the global Nipah cases, Bangladesh has not utilized antivirals or monoclonal antibodies on a compassionate basis.

## Why compassionate drug use is not enough

Past epidemics have demonstrated that without approved treatments, unregistered drugs are often used for emerging viral infections despite limited evidence of safety or efficacy. This approach fails to provide clinicians and policymakers with evidence-based treatment options. For instance, during the influenza A (H1N1)pdm09 pandemic, while at least 33,000 hospitalized influenza patients were treated “off label” with drugs not approved for use in severe influenza, fewer than 600 were enrolled registered clinical trials with publicly accessible results.^13^ Similarly, during the COVID-19 pandemic, convalescent plasma was widely administered without reliable evidence of efficacy, with over 100,000 patients in the United States receiving it under the Food and Drug Administration's Expanded Access Program.^14^ However, large-scale, rigorous randomized trials later showed that convalescent plasma did not improve survival or other clinical outcomes in hospitalized COVID-19 patients.^15^

Off-label drug use during outbreaks poses significant challenges because these treatments can become the ‘standard of care’ without robust evidence supporting their efficacy. This practice complicates, if not prevents, the testing of new therapies against these widely accepted yet unproven treatments, thereby perpetuating uncertainty regarding their true effectiveness, as observed with ribavirin for Lassa fever and oseltamivir for severe influenza.^13^^,^^16^^,^^17^ Therefore, the question arises: how can we build a solid evidence base for treating NiVD when cases are rare?

## Enhancing the evidence-base for clinical care of NiVD patients

To strengthen the evidence for NiVD care and effectively utilize the ongoing, albeit small and unpredictable, annual outbreaks, we propose focusing on four key areas: enhancing early case detection, optimizing supportive care to improve outcomes and establish a standard of care for trials, adopting a syndromic approach centered on encephalitis, and exploring innovative trials tailored to low case numbers as an alternative to ‘compassionate use’.

### Enhancing early case detection

Given that potential therapeutic agents, such as small-molecule antivirals, are most effective when administered early, timely detection of Nipah virus disease cases is essential for improving patient outcomes. Rapid case identification through robust surveillance—both sentinel and community-based—facilitates early intervention and clinical management, which is critical in reducing disease severity and mortality. Early detection also aids in implementing control measures, including contact tracing, isolation and quarantine, and infection control protocols.

Strengthening diagnostic, research, and clinical care capacities in rural hospitals, including essential infrastructure like cold-chain facilities, is crucial. Furthermore, investment in affordable, point-of-care diagnostic tools suited for peripheral settings will enhance timely case identification, support prompt enrollment in clinical trials, and improve access to interventions.

### Optimizing supportive care

There are not yet regionally-agreed best-practice guidelines for NiVD and practice is highly variable.^18^ The WHO developed evidence-based guidelines for Ebola virus disease to ensure optimal patient outcomes and facilitate reliable comparisons of new treatments in trials. Building on this approach, the WHO South-East Asia (SEARO) Regional Strategy for NiV (2023–2030) advocates establishing clinical management guidelines in affected countries and stresses the need for evidence-based supportive care for NiVD.^19^ These guidelines should also contain priority research questions to improve care standards. Additionally, training clinicians in standardized case management is crucial, particularly in areas with frequent NiV outbreaks, such as Kerala in India and the Nipah belt in Bangladesh.

### Adopting a syndromic approach to clinical management

Encephalitis, the primary clinical manifestation of NiVD, is prevalent in South and Southeast Asia, particularly in Bangladesh and India, where frequent outbreaks occur with unknown causes.^20^, ^21^, ^22^, ^23^, ^24^ Even when the cause is identified, the prognosis remains poor due to the lack of specific treatments.^25^^,^^26^ The recent Chandipura virus (CHPV) outbreak in India, with 245 cases and 82 deaths (case fatality ratio of 33%) between June–August 2024, underscores the potential benefits of a syndromic approach to managing encephalitis.^27^ CHPV, part of the *Rhabdoviridae* family, causes severe encephalitis, especially in children under 15.^28^

Given the challenges of conducting phase 3 NiVD-specific trials under current epidemiological and diagnostic conditions, a more practical approach would focus on encephalitis as a syndrome rather than exclusively on NiVD. By targeting encephalitis through trials that assess therapies for all-cause encephalitis, we can improve overall management and outcomes of patients presenting with encephalitis. A basket trial approach,^29^ which evaluates potential treatments across various encephalitis causes, would develop broader therapeutic strategies applicable to NiVD and other encephalitis etiologies. This dual focus offers a feasible solution to the NiVD's low incidence while enhancing encephalitis treatment overall.

However, evaluating treatments for encephalitis, including NiVD, requires clinical trials, which are challenging due to limited understanding of NiVD's clinical characteristics, pathogenesis, and current management practices. To address data gaps, we initiated a longitudinal cohort study in Bangladesh, a Nipah endemic country with annual outbreaks, enrolling patients with acute encephalitis syndrome, including NiVD, to detail demographics, clinical features, progression, treatment, etiologies, and outcomes. While this provides valuable insights in one region, the approach needs to be expanded. We propose adopting a standardized model similar to the International Severe Acute Respiratory and Emerging Infection Consortium (ISARIC)-World Health Organization (WHO) COVID-19 Clinical Characterization, using harmonized protocols, data tools, and a data-sharing platform.^30^ This model has proven effective in generating standardized data from multiple sites across the world, accessible for international research.

This network should be initiated in countries with a high burden of infectious encephalitis, particularly in the WHO South-East Asia Region (SEARO) and Western Pacific Region (WPRO), and expand to other countries at risk, including those with potential Nipah virus outbreaks. This approach will help generate standardized global data to optimize trial designs and improve outcomes for patients with acute encephalitis syndrome, including those with NiVD. Multilateral and multicountry collaborations among a diverse group of partners, including local regulators, researchers, and clinicians, would be critical to building such a network.

### Trials for virological outcomes

Traditional phase 3 clinical efficacy trials for evaluating potential treatments for NiVD are impractical due to the low number of NiVD cases– a modeling exercise for a cluster-randomized ring vaccination trial suggested it would take over 500 years to gather sufficient data under current epidemiological conditions.^31^

Pharmacometric trials offer a viable alternative by focusing on virological outcomes. Preclinical data from animal models support the evaluation of potential Nipah virus therapeutics, such as remdesivir, in human trials.^32^ Viral clearance can be quantified using Quantitative real-time PCR (qPCR) analysis of serial oral swabs after administering the candidate therapeutics, identifying which drugs effectively reduce viral load. This approach enables quick, cost-efficient prioritization of candidates for further testing, even with small sample sizes, given the current epidemiology of NiVD. Similar methods were employed in evaluating antivirals for COVID-19, helping to streamline the progression to clinical trials.^33^ Recent evidence has shown that viral clearance within the first five days of treatment serves as a surrogate marker for clinical efficacy in COVID-19.^34^ If this correlation holds for NiVD, this would support the use of viral clearance as an early-phase clinical trial endpoint to assess therapeutic efficacy.

Given the limited number of patients and trial sites, selecting and prioritizing drug candidates for clinical trials must be rigorous. The “core protocol” framework should be employed to facilitate patient recruitment across multiple sites and outbreaks, enhancing trial efficiency.^35^ Currently, animal model data support remdesivir and m102.4 for trials (either individually or in combination, for the prophylaxis and early treatment of NiVD), but m102.4 is not advancing; instead, the promising new mAb Hu1F5 is moving to a phase 1 trial.^36^^,^^37^

## Conclusion

The persistent threat of NiV in South and Southeast Asia underscores the need to enhance patient care. Compassionate drug use fails to generate the high-quality evidence required for informed clinical decision-making. A more pragmatic approach is needed, including standardized, evidence-based supportive care protocols and a syndromic approach that addresses encephalitis broadly, rather than focusing solely on NiVD, to improve the management of all encephalitis patients.

Innovative NiVD trials with virological outcomes over traditional clinical efficacy endpoints offer a feasible path for evaluating potential therapies, particularly with low case numbers. By integrating these strategies, healthcare systems in NiV-endemic regions will be better prepared to manage both current and future outbreaks.

## Contributors

MZH conceptualized the research problem, with AR, PO, and PH contributing to further developing the idea. MZH performed the literature review, led the analysis, and wrote and revised the initial draft. AR, PO, and PH provided supervision, feedback, and critical review of the scientific content, as well as editing of the draft. All authors reviewed and approved the final version of the manuscript.

## Declaration of interests

MZH is a Moh Family Foundation Fellow at the Pandemic Sciences Institute, University of Oxford, and is also supported by a scholarship through the Nuffield Department of Medicine, Clarendon Fund, and the Reuben Foundation. PH holds the Moh Family Foundation Professorship of Emerging Infections and Global Health at the Pandemic Sciences Institute, University of Oxford. AR, PO, and PH are supported by the UK Foreign, Commonwealth and Development Office, Wellcome [215091/Z/18/Z], and the Bill & Melinda Gates Foundation [OPP1209135]. The authors declared no conflicts of interest.
